# Exercise in People With Cancer: A Spotlight on Energy Regulation and Cachexia

**DOI:** 10.3389/fphys.2022.836804

**Published:** 2022-02-25

**Authors:** Jessica Allan, Linda A. Buss, Nick Draper, Margaret J. Currie

**Affiliations:** ^1^School of Health Sciences, Health and Human Development, University of Canterbury, Christchurch, New Zealand; ^2^Mackenzie Cancer Research Group, Department of Pathology and Biomedical Science, University of Otago, Christchurch, New Zealand; ^3^Department of Medicine, Christchurch Heart Institute, University of Otago, Christchurch, New Zealand

**Keywords:** exercise oncology, cancer cachexia, energetic capacity, energy regulation, inflammation

## Abstract

Exercise is increasingly becoming a standard of cancer care, with well-documented benefits for patients including improved mental wellbeing and reduced treatment-related side effects. However, important gaps in knowledge remain about how to optimise exercise prescription for people with cancer. Importantly, it remains unclear how exercise affects the progression of cancer cachexia (a wasting disease stemming from energy imbalance, and a common manifestation of advanced malignant disease), particularly once the condition has already developed. It was recently suggested that the anti-tumour effect of exercise might come from improved energetic capacity. Here, we highlight the possible effect of exercise on energetic capacity and energy regulation in the context of cancer, and how this might affect the progression of cancer cachexia. We suggest that due to the additional energy demand caused by the tumour and associated systemic inflammation, overreaching may occur more easily in people with cancer. Importantly, this could result in impaired anti-tumour immunity and/or the exacerbation of cancer cachexia. This highlights the importance of individualised exercise programs for people with cancer, with special consideration for the regulation of energy balance, ongoing monitoring and possible nutritional supplementation to support the increased energy demand caused by exercise.

## Introduction

Recently, the International Agency for Research on Cancer (IARC) provided an update on the global estimate of cancer incidence (GLOBOCAN) and estimated that in 2020 there were nearly 20 million new cases of cancer, inclusive of all sexes, ages, and cancer types (Ferlay et al., 2021). As cancer treatments advance, there are more people living with and beyond the disease. Exercise is becoming a prominent support strategy to attenuate many of the treatment-related side effects (Campbell et al., 2019). However, there are still many gaps in our understanding of exercise oncology, particularly with respect to how the underlying biological mechanisms affect response to therapy and patient outcome.

Early exercise trials pioneered change in cancer care, leading to a shift in the paradigm from a passive approach of bed rest toward an active approach of exercise (Winningham and MacVicar, 1988). Seminal work by Winningham and MacVicar (1988) established exercise as a care strategy for women with breast cancer who were undergoing adjuvant chemotherapy, which led to the development of the field of exercise oncology. Exercise oncology has continued to grow in popularity with benefits spanning further than care strategies into potential inhibitors of tumour growth, recurrence and mortality (Christensen et al., 2018).

A person’s energetic capacity can be compromised during cancer as energy is sequestered to fight emerging tumour growth (anti-tumour immunity and inflammation) and sustain existing tumours (Biro et al., 2020). This may be more apparent in people with advanced cancers due to numerous tumours and conditions such as cancer cachexia (a refractory wasting condition characterised by weight loss, and an indicator of energy deficiency).

Understanding the mechanisms involved in exercise oncology will enable exercise prescription to be more targetted and allow exercise oncology providers to create a more effective model of care for people with cancer (Jones, 2015). This mini-review focuses on the role of energetic capacity, cachexia, and immune function to identify potential implications for the individualised prescription of exercise for people with cancer.

## Immune Dysfunction and Energy Regulation in Cancer Cachexia

Cancer cachexia is a condition characterised by progressive body weight loss, which is accompanied by a decline in muscle strength, fatigue and anorexia (Reeves and Bernard, 2014). In addition, recent work suggests that cachexia is preceded by impaired systemic immunity (Ju et al., 2019). The syndrome is more common in people with advanced stage cancer, with some cancer types (such as pancreatic cancer) showing rates of as high as 85% (Henderson et al., 2018). Cancer cachexia is associated with increased mortality and there is currently no standard of care to improve or prevent the condition. Nutritional supplementation is sometimes used, but is not sufficient to treat the condition as a sole intervention (Solheim et al., 2018). Because exercise can improve muscle mass and strength, it is being investigated as a tool to prevent or treat cancer cachexia.

It is important to note that cachexia exists on a spectrum, starting with pre-cachexia (where muscle function may start to be impaired but is not yet clinically apparent), moving through to clinical cachexia, and finally to severe cachexia at end-stage disease (where the patient is largely bed-ridden). Two main sets of criteria for the diagnosis of cachexia exist: those by Fearon et al. (2011) and those by Evans et al. (2008). Those by Fearon et al. (2011) are based solely on weight loss, BMI and sarcopenia, while those by Evans et al. (2008) incorporate additional indicators such as abnormal biochemistry and fatigue. In both sets of criteria, a cut-off of 5% (involuntary) weight loss is used as the key determinant for the diagnosis of cachexia. Weight loss at this level is not necessarily extreme – in a person weighing 80 kg this is a loss of 4 kg over 6 or 12 months, depending on the criteria used. As such, exercise for people with cancer cachexia is likely to be feasible, particularly if the program is tailored to the individual [e.g., for someone with poor physical function, short walks and functional movements such as sit-to-stand exercises may be sufficient (Dittus et al., 2017)].

Cancer cachexia is characterised by systemic inflammation and immune dysfunction (Faber et al., 2009; VanderVeen et al., 2017). Similarly, overreaching (insufficient recovery from exercise) is characterised by high levels of circulating pro-inflammatory cytokines (Cheng et al., 2020) and impaired immunity, including lower cytokine production by myeloid cells, frequent upper respiratory tract illness, and changes in leukocyte subsets (Morgado et al., 2011; Peake et al., 2017b). Following exercise in healthy individuals, there is an influx of pro-inflammatory immune cells into the muscle (Peake et al., 2017a). These include pro-inflammatory (M1) macrophages, which interact with and promote the proliferation of satellite cells (Saclier et al., 2013). This is followed by a shift to an anti-inflammatory phenotype, which promotes tissue repair and muscle adaptation to exercise (Peake et al., 2017a). Here, M2 (anti-inflammatory or wound-healing) macrophages interact with differentiating satellite cells (Saclier et al., 2013). This sequence of events is highly temporally regulated, and if disrupted (e.g., by a prolonged inflammatory phase) will result in inefficient muscle adaptation (Tidball, 2017). In cancer cachexia, it may therefore be that due to the baseline level of inflammation being higher, there is an impaired capacity to resolve inflammation and muscle adaptation may be less efficient. A longer period of recovery may therefore be required between exercise bouts to allow resolution of exercise-induced inflammation in patients with pre-cachexia or cachexia. Alternatively, exercise intensity may need to be downregulated to avoid inducing excessive inflammation due to muscle damage. As much of our current knowledge regarding intramuscular macrophage response to exercise arises from muscle damage protocols, additional research is required to understand the macrophage response to varying intensities of training, such as low intensity exercise (non-damaging) and untrained populations completing low intensity exercise (potentially damaging) (Araujo Minari and Thomatieli-Santos, 2022). Further understanding of the inflammatory process post-exercise could allow for more targetted recovery strategies to be implemented.

In athletes, symptoms of training overload are often brought about or worsened by insufficient energy availability caused by inadequate energy intake (Stellingwerff et al., 2021). It is possible that in people with cancer (who may struggle to meet nutritional demands due to the high energy demand of the tumour and treatment-related nausea even when sedentary), nutritional supplementation may be required to support the additional energy demand from an exercise program. Of note, the MENAC trial is investigating the combination of exercise, nutrition and anti-inflammatory medication for the treatment of cancer cachexia, and will provide some information as to the effectiveness of such a multimodal intervention (Solheim et al., 2018).

**FIGURE 1:**
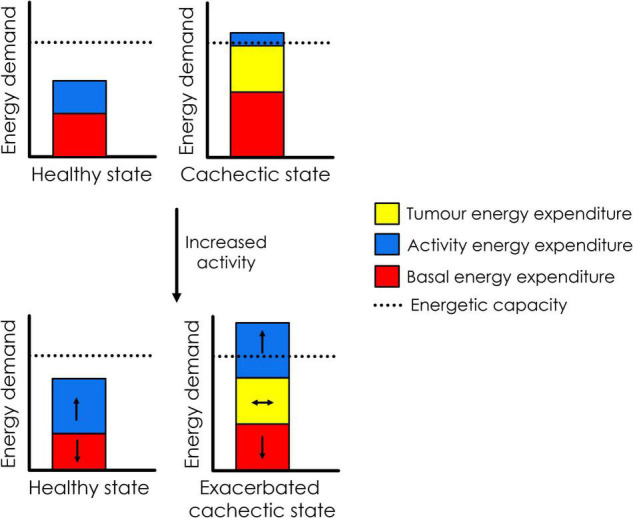
Hypothesised effect of increasing physical activity on energetics in patients with cancer cachexia. In the pictured, hypothetical scenario, increased physical activity results in a decrease in basal energy expenditure due to energy compensation. In healthy individuals, total energy expenditure remains well below energetic capacity. However, in the cachectic cancer patient (who is already in energy deficit due to high expenditure by the tumour and systemic inflammation, as part of basal energy expenditure), increased activity may result in downregulation of basal energy expenditure (possibly impairing anti-tumour immunity), while still pushing energy demand beyond energetic capacity. It is possible that this could exacerbate the cachectic state.

It is important to note that at its core, cancer cachexia is a syndrome stemming from energy imbalance and whole-body metabolic perturbations. It is difficult to predict how the body will respond to exercise in such a setting, given that there are currently few published clinical studies on the use of exercise in people with cancer cachexia (Grande et al., 2021). Most of our current knowledge comes from rodent models, in which exercise is almost always started prior to the development of cancer cachexia, and often long before tumour implant (Moreira et al., 2018; Ballarò et al., 2019; Niels et al., 2020). This means that the animals become conditioned to exercise before being presented with the large energetic challenge of a tumour, and energetic capacity would be expected to increase – thus priming the animal to cope better with the additional energy demands posed by the tumour. Indeed, exercise is usually beneficial in this setting [by slowing the onset of cachexia and maintaining muscle function for longer (Niels et al., 2020)]. However, the knowledge obtained from these studies may not be applicable to inactive patients who have already developed pre-cachexia or cachexia. As discussed in the next section, an additional energy demand in the form of exercise may actually exceed the individual’s energetic capacity and result in detrimental effects for patients with cachexia (Figure 1). Additionally, given that cachectic muscle suffers from a range of perturbations that affect its function (including mitochondrial dysfunction, and disruption of the balance between protein synthesis and degradation) (Rosa-Caldwell et al., 2020), it would perhaps be unwise to assume that exercise will be able to promote muscle growth and functional gain in the same way that it does in healthy muscle. Therefore, we suggest that caution and careful monitoring are warranted when implementing exercise programs that include patients with pre-cachexia or cancer cachexia.

## A Role for Energetic Capacity in Defining Tumour Response to Exercise

Recently, it was suggested that the anti-tumour effect of exercise might come from increased energetic capacity in trained individuals (Biro et al., 2020). Energetic capacity is defined as “the amount of energy that can be generated and used by an individual on a sustained basis” and is determined by both inherent (genetic) factors and modifiable factors such as training status (Biro et al., 2020). As such, energetic capacity can be increased by regular exercise training.

However, this situation is more complex in cancer, as the tumour itself can have a substantial additional energy demand on the body due to the uncontrolled nature of its growth (estimated to be between 100 and 1,400 kcal/day (Friesen et al., 2015). In addition, systemic inflammation resulting from tumour presence is associated with increases in energy expenditure of approximately 15% (Scott et al., 2001; Staal-van Den Brekel et al., 2016). These additional demands have been associated with the development of cancer cachexia which is in turn associated with increased mortality (Simons et al., 1999; Wu et al., 2013; Purcell et al., 2016). In such a setting, where energy demand exceeds energetic capacity, we suggest that it is possible that exercise might have detrimental rather than beneficial effects due to a further increased energy demand (if exercise is not prescribed carefully).

In support of this, we recently found that exercise which was started at the same time as tumour implant in mice resulted in reduced CD8^+^ T cell infiltration into the tumour, suggesting a reduction in anti-tumour immunity (Buss et al., 2021). We have also observed that most mice lose weight in the first few days after tumour implant, which is unlikely to be due to the development of cachexia as the tumour has not yet been established (Buss et al., 2020). We postulate that this is due to the dual challenge of exercise and tumour cell implant creating an ATP demand close to or exceeding what the mouse can sustain, leading to weight loss and impaired anti-tumour immunity. This could explain why *in vivo* studies show that exercise often has little effect on tumour growth when initiated post-tumour implant (Shewchuk et al., 1997; Jones et al., 2012; McCullough et al., 2013; Pedersen et al., 2016; Schadler et al., 2016), but can have substantial growth inhibitory effects when initiated prior to tumour implant (Pedersen et al., 2016). Exercise prior to tumour implant would be expected to increase energetic capacity, as the mouse is healthy and unchallenged by additional stressors, whereas the dual challenge of tumour induction (which would likely induce an immediate immune response, as well as energy costs to sustain the tumour as it becomes established) and exercise may create excessive energy demand.

Further evidence for the role of energetic availability in defining tumour growth and anti-tumour immunity comes from studies comparing mice housed below thermoneutrality (approx. 22°C) with those housed at thermoneutrality (approx. 30°C; thermoneutrality is the temperature zone in which a warm-blooded organism does not need to expend any energy on thermoregulation). In these studies, the authors found that tumour growth of four different transplantable tumours (B16-F10, 4T1, CT26, and Pan02) was significantly reduced (by approx. 100–600 mm^3^ at endpoint) by housing at thermoneutrality, and this was associated with increased proportions of CD8^+^ intratumoural T cells and decreased proportions of Foxp3^+^ intratumoural T cells (Kokolus et al., 2013). In addition, CD8^+^ cell depletion nullified the protective effect of thermoneutral housing, as did implant into immune-deficient mouse models (Kokolus et al., 2013). In a second study, thermoneutral housing enhanced the effect of anti-PD-1 treatment (Bucsek et al., 2017). These studies provide evidence that decreased energy expenditure (in this case on thermoregulation) can substantially improve anti-tumour immunity, presumably as there is more energy availability for immune responses.

With regards to exercise as a contributor to total energy expenditure, Careau et al. (2021) recently provided evidence that on increasing energy expenditure due to physical activity, total energy expenditure does not increase to an equivalent degree. Rather, basal energy expenditure (which includes energy expended on immunity) is downregulated. Similarly, mice do not show an additional increase in energy expenditure upon increasing wheel use (although there is an initial increase when the wheel is first introduced), and there is no correlation between the level of wheel use and energy expenditure across individual mice (O’Neal et al., 2017). It is therefore possible that in some individuals with cancer, a sudden increase in energy demand due to increased activity levels might have detrimental effects on anti-tumour immunity due to compensatory mechanisms to regulate total energy expenditure. Furthermore, we speculate that the threshold at which energetic capacity is exceeded is effectively lowered in people with cancer, due to the extra, unregulated energy demand created by the tumour. This means that people with cancer might need to exercise at lower volumes and/or intensities, monitor energy intake to ensure it is sufficient, and allow time for adequate recovery (lower exercise frequency) to obtain benefit while avoiding overreaching.

## Current Exercise Oncology Guidelines

In 2018, leading international exercise oncology researchers convened to update the American College of Sport Medicine (ACSM) guidelines for people with cancer. The ACSM guidelines recognise there is strong evidence that exercise supports people with cancer by improving symptoms of cancer-related fatigue, cancer-related depression and anxiety, health-related quality of life, physical function and lymphoedema (Campbell et al., 2019). The researchers provided exercise prescription recommendations that vary based on the specific negative side-effects experienced (Campbell et al., 2019). The majority of these recommendations involve combined resistance and aerobic exercises of moderate to vigorous intensity, two to three times per week for 12 weeks (Campbell et al., 2019).

Researchers agree that no one prescription will suit all people with cancer, and individualisation is paramount due to the complexities of cancer and cancer treatment (Campbell et al., 2019; Stout et al., 2020). Stout et al. (2020) have highlighted that people with cancer vary greatly in their affinity for exercise, which can be dictated by side-effects from treatment, previous exercise history, environmental constraints and safety concerns. Furthermore, Campbell et al. recognised limitations to their ACSM guidelines and emphasised caution when interpreting and applying an exercise prescription. The authors highlighted that the majority of available evidence in safety and efficacy of exercise has been based on randomised control trials in breast cancer survivors (Campbell et al., 2019), which has a comparatively low incidence of cachexia (Baracos et al., 2018). This is an important consideration when prescribing exercise to people with advanced stage cancer and cancer cachexia, who may have limited available energetic capacity and/or impaired immune function.

The ACSM guidelines report by Campbell et al. (2019), and The National Comprehensive Cancer Network (NCCN) recommend that people with advanced cancer should have a pre-exercise medical evaluation and be referred to exercise professionals. Researchers have emphasised that although the ACSM guidelines vary depending on presenting side-effects, they are not tailored to a person’s starting exercise capacity (Carter et al., 2021). The nature of cancer and cancer treatments causes people’s exercise capacity during treatment to vary greatly and the guidelines need to be viewed as a goal and not as an achievable place for people to commence exercise (Stout et al., 2020). Clinical evaluation of objectively measured exercise capacity as a starting point can assist to individualise exercise prescription and dictate the upper limits of exercise (Carter et al., 2021).

## Exercise Prescription for People With Energy Deficiency or Cancer Cachexia

A 2021 Cochrane review by Grande et al. (2021) reviewed the evidence of exercise for cancer cachexia in adults and highlighted the limited research in this population. It included four studies, which encompassed cancers of head and neck (Capozzi et al., 2012, 2016; Grote et al., 2018), lung and pancreas (Solheim et al., 2017), and mixed (Forget et al., 2014). Grande et al. (2021) acknowledged that there is insufficient research to determine the effectiveness, acceptability, and safety of exercise for adults with cancer cachexia. However, it has been suggested that 50–80% of people with advanced cancer experience cachexia, which emphasises the need for caution when prescribing exercise based on the ACSM guidelines (Argilés et al., 2014). Further research for people with cancer cachexia is required to develop safety and efficacy regulations, and to provide a deeper understanding of the biological mechanisms involved during exercise for people with cancer cachexia.

Due to the energy demands of exercise and the potentially limited availability of energy in people with advanced cancer and cancer cachexia, exercise needs to be carefully prescribed to avoid detriment. Understanding the role of energetic capacity in defining the tumour response to exercise could elucidate further recommendations around the frequency and timing of exercise for people experiencing energy deficiency. Mouse studies have demonstrated that the timing of exercise can play a critical role in its effectiveness (Pedersen et al., 2016; Eschke et al., 2019), suggesting that exercise may only be beneficial when prescribed to a person with sufficient energetic capacity. Therefore, determining an individual’s energetic capacity before commencing an exercise intervention, and regular monitoring of energetic capacity may be critical. Implementing sub-maximal cardiorespiratory testing can provide exercise practitioners with estimated oxygen availability, and aerobic threshold testing could provide further insight into an individual’s oxidative capacities. Furthermore, collecting complete blood counts and measures of circulating inflammatory cytokines could elucidate the patient’s inflammatory status and immune function, and assist in guiding the exercise prescription. Monitoring recovery and aligning recovery with biomarker analysis could provide further insight into the frequency, intensity, time and type of exercise interventions with most benefit (Grote et al., 2018).

## Future Research Implications and Conclusion

The aim of this mini review was to highlight the potential effects of energy regulation and cancer cachexia when prescribing exercise for people with cancer. Clinicians and researchers alike are supportive of individualisation of exercise prescription during cancer care - however, minimal research has been completed for people with cancer cachexia (Grande et al., 2021). There are potential considerations for exercise within this population that could help regulate exercise by the F.I.T.T (Frequency, Intensity, Timing, and Type) principle, including submaximal exercise testing and monitoring alongside biomarker analysis.

While exercise has been shown to have many benefits for people with cancer, gaps in knowledge remain. In particular, it is unclear how patients with cancer cachexia cope with an increase in exercise. Current knowledge supporting the use of exercise to manage cancer cachexia comes from rodent studies and limited trials in humans. We speculate that people with cancer might be more prone to overreaching and lowered immunity due to the additional energetic demand caused by the tumour, particularly once cachexia has developed. Given the many benefits that exercise can provide for people with cancer (e.g., reduction in treatment-related side effects, improved mental well-being), we do not suggest that exercise should be avoided. However, we suggest that energetic regulation with increasing exercise, particularly in the context of cancer cachexia, is an important avenue for future research to establish the safety of exercise in people with the condition. We reiterate that it is essential for exercise prescription for cancer patients to occur on an individualised basis, with appropriate nutritional support if required.

## Author Contributions

JA, LB, and MC: manuscript conception. JA and LB: writing – initial draft. ND and MC: supervision. All authors edited and wrote the review, contributed to the article, and approved the submitted version.

## Conflict of Interest

The authors declare that the research was conducted in the absence of any commercial or financial relationships that could be construed as a potential conflict of interest.

## Publisher’s Note

All claims expressed in this article are solely those of the authors and do not necessarily represent those of their affiliated organizations, or those of the publisher, the editors and the reviewers. Any product that may be evaluated in this article, or claim that may be made by its manufacturer, is not guaranteed or endorsed by the publisher.
